# Implementing recommended falls prevention practices for older patients in hospitals in England: a realist evaluation

**DOI:** 10.1136/bmjopen-2025-099698

**Published:** 2025-12-14

**Authors:** Natasha Alvarado, Lynn McVey, Frances Healey, Dawn Dowding, Hadar Zaman, V-Lin Cheong, Peter Gardner, Alison Lynch, Nick Hardiker, Rebecca Randell

**Affiliations:** 1 University of Bradford, Bradford, UK; 2 University of Leeds, Leeds, UK; 3 The University of Manchester, Manchester, UK; 4 Leeds Teaching Hospitals NHS Trust, Leeds, UK; 5 Manchester University NHS Foundation Trust, Manchester, UK; 6 University of Huddersfield, Huddersfield, UK

**Keywords:** Risk management, Hospitals, GERIATRIC MEDICINE, QUALITATIVE RESEARCH, PREVENTIVE MEDICINE

## Abstract

**Abstract:**

**Objective:**

To explore why there is variation in implementation of multifactorial falls prevention practices that are recommended to reduce falls risks for older patients in hospital.

**Design:**

Mixed method, realist evaluation.

**Setting:**

Three older persons and three orthopaedic wards in acute hospitals in England.

**Participants:**

Healthcare professionals, including nurses, therapists and doctors (n=40), and patients aged 65 and over, and carers (n=31).

**Intervention:**

We examined mechanisms hypothesised to underpin the implementation of multifactorial falls risk assessment and multidomain, personalised prevention plans.

**Primary and secondary outcome measures:**

We developed an explanation detailing that how contextual factors supported or constrained implementation of recommended falls prevention practices.

**Results:**

Nurses led delivery of falls risk assessment and prevention planning using their organisation’s electronic health records (EHR) to guide and document these practices. Implementation of recommended practices was influenced by (1) organisational EHR systems that differed in falls risk assessment items they included, (2) competing priorities on nurse time that could reduce falls risk assessment to a tick box exercise, encourage ‘blanket’ rather than tailored interventions and that constrained nurse time with patients to personalise prevention plans and (3) established but not recommended falls prevention practices, such as risk screening, that focused multidisciplinary communication on patients screened as at high risk of falls and that emphasised nursing, rather than Multidisciplinary Team (MDT), responsibility for preventing falls through constant patient supervision.

**Conclusions:**

To promote consistent delivery of multifactorial falls prevention practices, and to help ease the nursing burden, organisations should consider how electronic systems and established ward-based practices can be reconfigured to support greater multidisciplinary staff and patient and carer involvement in modification of individual falls risks.

**STRENGTHS AND LIMITATIONS OF THIS STUDY**

- Multiple methods were used in three organisations that enabled a rich, detailed explanation to be developed about how contextual factors influence implementation of falls prevention practices.
- Participants included multidisciplinary staff, patients and carers who provided diverse perspectives and insight into falls prevention in hospital.
- The four mechanisms investigated reflect the complex and multifaceted nature of falls prevention and were prioritised by stakeholders, including patient and public representatives.
- It would be beneficial to explore in greater detail multidisciplinary documentation systems and the delivery and impact of specific interventions intended to modify falls risks.

## Introduction

 Falls, defined as events that result in a person coming to rest inadvertently on the ground or floor or other lower level, are a leading cause of morbidity and mortality globally.^1 2^ In hospitals in the UK, falls are the most commonly recorded safety incident, with over 240 000 inpatient falls reported annually in England and Wales.^3^ Around a third of falls result in physical injuries and 1%–3% in fractures.^3 4^ Falls in hospital are a particular concern for older people who are at greater risk of falling due to age-related physiological changes, such as gait instability and changes in vision^5 6^ and who are more likely to incur physical injury from falls, including fractures, contusion, laceration, brain injury and death.^7 8^ Even where physical injury is minimal, there can be psychological harm to patients, carers and staff, including distress and loss of confidence that may constrain patient recovery.^2 4^ An unfamiliar hospital environment further increases the risk of older people falling^4^ and there are financial implications for healthcare organisations through extended hospital stay and resource use.^9 10^ The cost to the National Health Service (NHS) of inpatient falls is estimated at £630 million annually.^11^

While the strength and quality of evidence are limited, systematic reviews have indicated that multifactorial approaches, where falls risk assessment and prevention planning link individual falls risks to appropriate interventions, may reduce falls in hospital.^12^,^14^ These studies have informed clinical guidance for falls prevention nationally and internationally.^2 15^ In England, the National Institute for Health and Care Excellence (NICE) Clinical Guidance for falls prevention (CG161) advises that patients aged 65 and older, and patients aged 50–64 and judged by a clinician to be at higher risk of falling, receive a multifactorial falls risk assessment on admission to hospital and multidomain, personalised interventions (CG161 was in place at the time of the study, since updated as NICE CG 249). Nine domains are suggested to assess individual falls risks, see box 1.

Box 1 **Individual falls risk assessment domains**

1. Cognitive impairment.
2. Continence.
3. Fall history.
4. Footwear.
5. Health problems.
6. Medication.
7. Postural instability, mobility and/or balance problems.
8. Syncope syndrome.
9. Visual impairment.

A clear distinction can be made between multifactorial falls risk assessment and falls risk screening.^16^ Falls risk screening typically involves falls risk assessment to score and stratify patients as high, medium or low risk of falls, with standardised, rather than personalised, interventions delivered depending on the level of risk.^17^ There is little evidence that risk screening helps reduce falls in hospital.^18^ Studies have shown that discontinuing risk screening does not increase risk of falls^19 20^ and this practice is not recommended for falls prevention in hospital.^2 15^ Instead, the multifactorial approach seeks to identify and modify individual falls risks and requires multidisciplinary input, for example, pharmacists to review, reduce or discontinue medication that may be causing a falls risk and physiotherapists to address issues with mobility or balance problems through rehabilitation. Additionally, patient and carer involvement is necessary to personalise prevention plans.

Despite national guidelines and quality improvement initiatives, falls remain a persistent safety challenge in hospitals and, while multifactorial falls risk assessment and multidomain, personalised interventions are recommended, there is substantial variation between hospitals in their implementation, for example, in level of falls risk assessment and intervention delivery.^11^ The reasons for this variation have not been explored. Therefore, this study investigated how, why and the extent to which recommended falls prevention practices were translated into ward-based practices to reduce falls risks for older people and what supported and constrained the implementation process.

## Methods

Realist evaluation was used to address the research questions; this approach involves constructing, testing and refining programme theory,^21^ that is, explanation of how and why a programme or intervention is expected to produce its intended outcome.^22^ Realist evaluation provided a useful study framework because context is key in realist explanation; outcomes (programme impacts) are considered dependent on the context in which the programme is introduced.^21^ The programme may produce the desired impact in one context but not in others and it is these eventualities that realist evaluation seeks to explain. To focus the investigation, realist evaluation offers the concepts of Context+Mechanism = Outcome (CMO). Mechanisms explain how and why individuals or groups respond to programme resources that generate change. Contexts are the circumstances that influence (support and constrain) individual or group responses to programme resources, such as individual attitudes and beliefs, workplace cultures and routines. Outcomes are the intended and unintended impacts of interactions between contexts and mechanisms.

**Figure 1:**
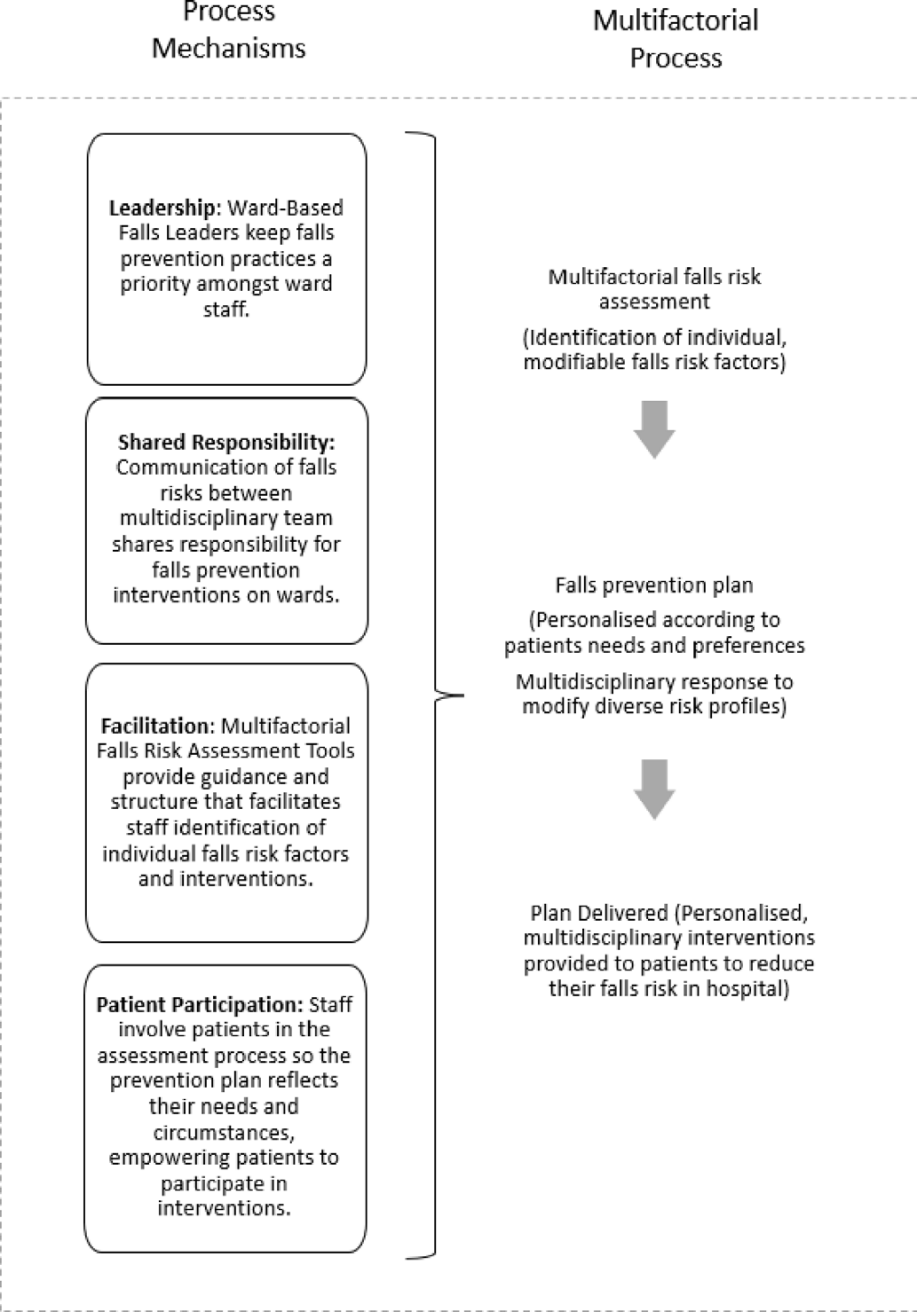
Programme theory for delivery of multifactorial prevention practices.

For this study, four mechanisms were theorised to underpin the implementation of multifactorial falls prevention practices, namely leadership, facilitation, shared responsibility and patient participation (see figure 1). These were derived from a realist review of the literature and configured in a programme theory and as CMOs.^23^ The mechanisms were used to guide data collection and analysis in this multisite case study where ethnographic observations, interviews with healthcare staff and patients and carers and a review of patient electronic health records (EHRs) were conducted to investigate how circumstances supported and constrained the mechanisms and implementation of falls prevention practices.

### Study setting

Case study sites were chosen for their potential to test and refine the programme mechanisms.^21^ Data were collected in three older person and three orthopaedic wards, as the intention was to capture the care of patients targeted by NICE CG161, that is, patients aged 65 and older or between 50 and 64 and judged to be at higher risk of falling. One of each type of ward was studied within three NHS organisations in England.

### Study sample and data collection

In realist evaluation, sample size is determined by theoretical considerations; the adequacy of data is judged by its depth and diversity to test and refine the mechanisms under investigation.^21 24^ To ensure adequate depth and diversity of data, we conducted observations and interviews as follows.

#### Observations

Observations aimed to capture events and interactions related to delivery of falls risk assessment and prevention practices. Observations were conducted in 4-h blocks in each ward and took place at different times of day, including night shifts, and different days, including weekends. Researchers used an observation schedule with prompts that reminded them to capture details about each mechanism: leadership, facilitation, shared responsibility and patient participation. For example, in relation to leadership, the schedule included prompts for researchers to document what staff was observed to do in ward-based falls leadership roles, and for shared responsibility, how falls risk information was communicated between the multidisciplinary team in team meetings and informally. Data collection took place between November 2021 and June 2023 and was conducted by three members of the research team (NA, LM and RR) who each independently observed every ward at least once.

#### Interviews

There is no consensus on the optimal number of qualitative interviews.^25 26^ For this realist evaluation, we used a purposive sampling strategy to capture the experiences and perspectives of key stakeholders relevant to programme theory testing, namely ward staff, patients’ and carers. For staff, we sought to capture multidisciplinary perspectives and experiences, for example, nurses, healthcare assistants and support workers, doctors and therapists. For patients, we aimed to recruit individuals with different falls risks (as advised by staff) and who were aged 65 and older, or between 50 and 64 and judged to be at a higher risk of falls, and their carers (where present). Patients were also asked for their permission to review their falls risk assessment and prevention plan documentation. Interviews were conducted and recorded *via* MS Teams or on the ward, using a digital recorder, and took the form of the realist teacher/learner cycle, whereby researchers explained each realist CMO hypothesis to the interviewee, who was asked to discuss if the explanation resonated with their experience, ‘teaching’ the researcher about what happens in practice.^27^ Anonymised data from patients’ falls risk assessment and prevention plans were extracted from EHRs into an MS Excel spreadsheet. Recruitment continued until the team judged that they had sufficient data (representing a range of stakeholder experiences) to refine their CMO explanations.

### Analysis

The outcome of interest was implementation of multifactorial falls prevention practices; this was operationalised using patients’ EHR data to confirm if participants had received a falls risk assessment (to identify their individual falls risk factors) and a falls prevention plan (to link individual risks with multidomain, personalised interventions). The assessment documentation was reviewed to understand how it was structured and the falls risk items included as prompts. The data extraction form recorded if a falls risk assessment and prevention plan was completed by assessing if all risk assessment and prevention plan items had a response documented against them. Descriptive statistics were prepared to summarise the data by organisation.

Framework analysis was used to analyse interview and observation data.^28^ This approach involved researchers familiarising themselves with the data by reading a selection of anonymised interview and observation transcripts. They then came together to discuss their interpretations and to construct a thematic framework that was used to index (catalogue) the data using NVivo (qualitative data analysis software). Indexed data were summarised in data matrices that grouped themes relevant to each mechanism: leadership, facilitation, shared responsibility and patient participation. Data were compared against the mechanism to understand if findings supported or refuted the explanation and to understand how contextual factors influenced the mechanisms at work.

### Patient and public involvement

A Lay Research Group (LRG) (a group of service users and carers who had either fallen themselves or cared for someone who fell in hospital) provided advice and support throughout the study. An LRG representative was a member of the project management group and contributed to the development of the project proposal. The LRG group helped prioritise the mechanisms that focused the investigation; they contributed to the development of the observation protocol and interview topic guides for patients and carers, provided a patient perspective on the qualitative data analysis, and coauthored a paper on how patient and public and research groups can work together collaboratively and successfully.^29^

## Results

In total, researchers conducted 40 interviews with ward staff, including 14 ward nurses, 7 ward managers, 5 healthcare assistants, 5 doctors, 4 physiotherapists, 2 pharmacists, 2 support staff and 1 occupational therapist, and interviews with 28 patients and 3 carers, see table 1 for summary of data collection by organisation.

**Table 1:** Summary of data collected by organisation

Organisation	Hours of observation	Staff interviews	Patient and carer interviews	EHR review
1	89	15	10	20
2	82.25	13	10	20
3	80	12	11	20
Total	**251.25**	**40**	**31**	**60**

EHR, electronic health record.

### Organisational falls prevention policy

All organisations’ falls prevention policies mandated a multifactorial falls risk assessment for all patients aged 65 and older and for patients aged 50–65 and judged to be at higher risk of falling. These practices were monitored through audit (national and local) and root cause analyses were performed when a fall with patient harm was reported. Falls risk assessment documentation—including a falls risk assessment tool and prevention plan—was digitised in the EHR in each organisation and embedded in nursing documentation, that is, nurses were responsible for assessing patients’ falls risks and documenting their prevention plan. The EHR review, see table 2, indicated that wards complied with hospital policy, with prevention plans often updated on the night shift.

**Table 2:** Summary of EHR review data

Organisation	Falls risk assessment documented (per site, n=20)	Falls prevention plan documented on day shift	Falls prevention plan documented on night shift	Falls prevention plan documented (per site, n=20)
1	20	11	9	20
2	20	4	16	20
3	19	7	13	20
Total	**59**	**21**	**39**	**60**

EHR, electronic health record.

### Fall risk assessment documentation

Table 2 suggests that the practices of interest in this study were implemented. However, organisations had different EHRs, developed either in-house or by external providers, and therefore, the content (structure and items) of falls risk assessment tools differed by organisation. We compared risk items in each tool against the nine domains suggested by NICE (CG161), see table 3.

**Table 3:** Falls risk assessment items by organisation/documentation tool

NICE CG161	Organisation		
	1	2	3
Cognitive impairment	A/PP	A	A
Continence	PP	A	N
Fall history	A	A	A
Footwear	PP	?	PP
Health problems	N	A	N
Medication	N	A	PP
Postural instability, mobility and/or balance problems	A/PP	A	A
Syncope syndrome	PP	N	?
Visual impairment	PP	A	N

?, unable to identify in falls risk assessment or nursing assessments; A, explicitly included in falls risk assessment; CG161, clinical guidance for falls prevention; N, identified in other nursing assessments; NICE, National Institute for Health and Care Excellence; PP, included in falls prevention plan.

Table 3 demonstrates that falls risk assessment documentation included multiple risks, but the extent to which they explicitly encompassed the NICE domains as prompts in the assessment and care plan differed, pointing to how such tools may create variation in what is routinely assessed and documented across organisations. Additionally, practice observations and participant interviews indicated that established falls prevention practices and competing priorities on nurse time influenced the mechanisms—leadership, shared responsibility, facilitation and patient participation.

### Established falls prevention practices

Established falls prevention practices were found to influence the leadership of falls prevention practices and how responsibility for falls prevention was shared on the ward.

#### Leadership of falls prevention practices

In day-to-day practice, nurses led delivery of the falls risk assessment and prevention plan. An established practice, alongside falls risk assessment, was screening to identify patients considered at high risk of falls (not recommended for falls prevention in hospital). This was also led by nurses. In organisation 3, patients’ risk was automatically scored in the EHR according to responses to the falls risk items, history of falls, mobility and cognitive impairment. In organisation 2, nurses manually scored patient risk to allocate enhanced observations of care. Screening tools were not used in organisation 1, but staff identified and discussed patients considered to be at ‘high risk’ of falls using their clinical judgement.

#### Enhanced patient supervision

Risk screening was typically used to allocate interventions based on ‘enhanced patient supervision’, for example, one-to-one care or being placed on a cohort bay, where a staff member—typically a nurse or healthcare assistant—stayed in a bay (usually consisting of four beds) to observe patients and reduce their risk of falling by providing direct assistance. A physiotherapist explained why a patient might receive enhanced supervision.

If they’re (a patient) independently mobile, you can’t fix them anywhere but like I say, they’re restless and walking up and down the ward and they’re at risk of fall by the nature of them being confused or delirious and the fact that they’re constantly walking up and down and depending on caseload (…) we can be referred or asked and say ‘They’re a falls risk’ but at least from a physiotherapy point of view, well they’re good on their feet, it’s more the level of confusion putting them at risk (…) which is why they might need someone one-to-one with them. (Physiotherapist, Older Person Ward, Organisation 1)

Many patients on the wards studied had some level of cognitive impairment that could be temporary, for example, due to infection or postsurgery or a longer term condition, such as dementia. These patients could become confused, agitated and engage in behaviours referred to as ‘wandering’ or ‘walking with purpose’ that manifested predictably, at certain times, for some patients and unpredictably for others. These behaviours often meant that the patient was screened as at high risk of falls and allocated enhanced patient supervision. Use of supervision as a key intervention placed emphasis on nurses and healthcare assistants as responsible for falls prevention. A physician commented:

‘I think falls in hospital (…) is probably considered to be a supervision issue (…), if it’s a medical factor that’s causing somebody to fall, so, for example, a blackout (…) I think medical staff will get involved with that. But I think the majority of the time in terms of reducing falls on the ward, I think probably it is—maybe not on paper or even consciously—but predominantly left with nursing staff, I would say’. (Physician, Older Person Ward, Organisation 3)

The participant highlights that the responsibility for falls prevention is ‘*predominantly’* left with nursing staff. However, a challenge for nursing teams was that often wards were not staffed to deliver enhanced supervision in line with identified need. Conversations with healthcare assistants during observations indicated the impact on them of delivering supervision.

The healthcare assistant says it feels like they put people on one-to-one and falls (cohort) bay and you’re (the healthcare assistant) expected to look after them all, but you can’t; you can’t watch someone if you’re looking after someone else at the same time. A while back they were meant to have two staff on the falls bay: one to watch and one to care. But it didn’t work for staff because the staff who was meant to care had to do everything and they were pulling their backs out trying to move patients on their own. (Observation, Orthopaedic Ward Organisation 1, paraphrasing a Healthcare Assistant)

The healthcare assistant explained the difficulty of preventing falls when having to both assist and supervise up to four patients simultaneously; they described it as mentally and physically draining.

#### Shared responsibility for falls prevention

The expectation that supervision would prevent falls was also implicit in multidisciplinary communication about falls risks. Interviewees explained that information about patient care was shared verbally (non-nursing staff reported that they did not typically refer to or use the falls risk assessment documentation used by nurses), with formal communication taking place in multidisciplinary team meetings, such as daily ‘safety huddles’. Observations of these meetings indicated that typically it was patients screened at high risk of falls who were discussed by the team and supervision-based interventions. Multidisciplinary communication about falls risk is discussed further in a separate paper.^30^ While formal communication appeared to focus on patients screened as at high risk of falls, physicians, therapists and pharmacists explained how they considered falls prevention for individual patients, for example, physiotherapists on orthopaedic wards assessed all patients and decided how they should mobilise safely, and a pharmacist reported that they typically review the medication of all older patients on admission to hospital and commented:

I know we’ve done some sort of looking at benzodiazepines (sedative medication) and reducing them down, (…). But then we’ve had some people where we needed to continue those because just the risk of withdrawal’s probably too high. I suppose it’s taken in the context of, I suppose, looking to see if the patient’s got delirium or any other problems. (Pharmacist, Older Person Ward, Organisation 2)

The pharmacist explained that when deciding whether to make a change in medication, they must consider side effects, such as withdrawal and differences in opinion with physicians. Therefore, medications that cause falls risks might not be quickly modified during a patient’s hospital stay, with ward staff, typically nurses and healthcare assistants, having to manage the ongoing effects of the risk through supervision.

In summary, established falls prevention practices consisted of nurses leading a hybrid approach to falls prevention that combined falls risk assessment and risk screening. Use of risk screening and associated interventions based on patient supervision shaped multidisciplinary communication about falls risk and placed emphasis on nurses and healthcare assistants as responsible for preventing falls through constant patient supervision.

### Competing priorities and workload pressure

Competing priorities and workload pressure were observed to influence how and the extent to which (1) falls risk assessment documentation facilitated delivery of recommended practices and (2) patients participated in falls prevention.

#### Use of electronic falls risk assessment documentation

Nurses were responsible for assessing and documenting many risks alongside falls, for example, pressure ulcers, hygiene and continence. Regarding the amount of assessment documentation, a nurse commented ‘*that’s* (documenting practice) *more of the nursing role now than actually delivering the care*’ (*Nurse, Older Person Ward, Organisation 3*) and explained that time spent documenting practice constrained their time with patients. Conversations with, and observations of, nurses indicated that documentation was completed when they found the time and opportunity to use the EHR, providing insight into why prevention plans were often documented on the night shift. In this context, a nurse explained how assessment tools could facilitate identification and modification of individual falls risks.

It (assessment tool) kind of reminds you of certain things that you probably are not thinking straight away, you know, has that patient got on non-slip socks? (Nurse, Older Person Ward, Organisation 3).

In this organisation, footwear was one of the two items identified in the prevention planning documentation and was said to act as a useful reminder if the risk had not been assessed in earlier interactions with patients. However, a nurse in a different organisation discussed:

I think it’s (EHR) really good risk assessment (…), but I think we’ve put lots of things in that are blanket for everyone and then we’re not able to then deviate away from that. I don’t think staff feel necessarily empowered to deviate away from it. I think staff are concerned about if, for example, the risk assessment said they needed a cohort bay and then they didn’t put them in a cohort bay and then they had a fall, about how that might affect them. (Matron, Organisation 1, Orthopaedic Ward)

This organisation’s falls prevention plan listed seven items in the form of questions with suggested interventions, for example, ‘*Is the patient in the most appropriate place for their needs? For example, close to nurse station, close to the toilet?*’ The nurse explained that workload pressure may encourage a ‘*task orientated*’ approach, meaning that, rather than personalising interventions based on a patient’s needs, nurses may not deviate away from those suggested by the assessment tool in case a fall occurs. Additionally, a nurse commented:

It’s really easy to do a tick-box, and then get side-tracked with something else and not do anything with that information. (Nurse Manager, Older Person Ward, Organisation 1).

While assessment tools facilitated systematic documentation of falls risks, they may encourage a ‘tick box’ approach, that is, where what is documented differs from what happened in practice. Further examples of a tick box approach when using falls risk assessment tools were observed; a nurse explained that retrospective documentation may not reflect the full extent of care delivered due to memory limitations, and one organisation’s EHR enabled ‘cutting and pasting’ from one record to another. While this function could save nurse time, observations captured that some prevention plans were not updated in ways that accurately reflected patient progress or changes in their care. Even so, falls risk assessment documentation was completed as a matter of habit, a nurse explained:

If there’s a complaint, or there’s ever anything that somebody wants to question what you’ve done on that shift, for whatever reason, then that’s your evidence. Because the nurses’ old chestnut: if it isn’t written down it hasn’t happened (Senior nurse, Orthopaedic Ward, Organisation 3).

Documentation provided protection against complaints or questioning regarding falls incidents.

#### Patient participation in falls prevention

Competing priorities on nurse time also influenced patient participation in falls prevention. Observations and interviews indicated that risk assessment was a dynamic process, undertaken by nurses using a combination of observation, conversations with patients and carers and the EHR, but patients were not observed to be directly involved in the formal falls risk assessment or prevention planning process. While nurse time with patients was constrained by competing priorities, such as care documentation, other staff, including physiotherapists and carers/visitors, were able to elicit and share information that could help personalise care. Two organisations employed engagement workers—non-clinical staff available to talk to patients about their needs, interests and hobbies. Additionally, all organisations had developed documents designed to help staff ‘get to know their patients’. A nurse explained:

It’s so important (understanding patient perspectives), and I've had the opportunity to spend 20 min on the phone with a family member, filling that (Getting to Know Me document) in and getting to know that patient and saying, “Well, no wonder they're trying to get up to get a coffee. We've given him a cup of tea.” Even that is so basic, so simple. If we all just knew that, stop giving him a cup of tea, let him have a coffee with his paper, he wouldn't try to get up. He wouldn't think he’s at home. (Senior nurse, Orthopaedic Ward Organisation 2)

These initiatives recognised the importance of incorporating patient perspectives and preferences in care delivery.

Although patients were not observed to be involved directly in completing the falls risk assessment or prevention plan, observations and interviews revealed routine ways in which nurses and other ward staff encouraged patients to participate were *via* their use of the call bell if they needed assistance mobilising and participation in their rehabilitation programmes. In these interactions, skills, such as persuasion, compassion, patience, sincerity and reassurance, were described by staff and patients to support whether patients acted on such messaging. However, a patient explained:

I try never to (walk) without somebody being with me, but sometimes you’ve got to be a bit naughty (…) when you’re ringing and ringing and you’ve got nowhere else to go and you think to yourself, “Oh, Lord, I’m going to make a mess*”* (Patient, Orthopaedic ward, Organisation 2).

Patients did not always receive a timely response to their use of the call bell, circumstances that may lead them to mobilise alone if they do not feel able to wait for assistance when urgently needing the toilet. Similarly, regarding rehabilitation, a senior support worker discussed:

You’ve got a good patient that wants to (…) follow physio’s instructions but, oh, can you just wait, (…). I’m busy, and then they get up because they’re probably fed up of waiting or the physios are telling them you need to do it and they get up without that supervision and then (…) God forbid, they have an accident. (Senior Support Worker, Older Persons Ward, Organisation 2)

Wards were resourced differently, for example, in one organisation, the orthopaedic ward had a dedicated therapy team who were located on the ward. In comparison, the older person ward in the same organisation had physiotherapists who visited the ward at certain times and worked across several wards. These circumstances impacted the workload of staff and consequently their time to support patient participation in rehabilitation programmes and their availability to respond to requests for assistance.

In summary, falls risk assessment tools facilitated documentation of falls prevention practices, but competing priorities on nurse time constrained the extent to which these tools supported personalisation of prevention plans and the extent to which they accurately reflected care delivery. Nurses sought to understand patients’ needs and preferences outside the formal falls risk assessment with support from colleagues and families and carers, and provided patient-directed safety messaging in care delivery. Whether patients acted on such messaging was influenced by staff interactional skills and their availability to respond to patients’ requests for help.

## Discussion

This study explored if, how and to what extent mechanisms labelled leadership, shared responsibility, facilitation and patient participation underpinned implementation of multifactorial falls risk assessment and personalised, multidomain, prevention plans and the circumstances that influenced the mechanisms at work. Previous studies have explored barriers and facilitators to the use of clinical guidelines by healthcare staff,^31^,^33^ with several common factors highlighted, for example, characteristics of the guideline itself and individuals’ awareness and knowledge of the guideline. In this study, we have configured an explanation about how such factors interact to shape implementation of recommended practices. In the following discussion, it should be noted that, in addition to the multifactorial falls risk assessment process, we observed individual interventions embedded in care which guidelines find little or no evidence to support, such as use of nonslip socks.^34^

The findings showed that recommendations from clinical guidelines were reflected in organisational policy, and, at practice level, falls risk assessment documentation was provided in the EHR to support risk identification. Assessment documentation was completed as a matter of habit, partly as protection against complaints and interrogation—‘*the nurses old chestnut’*. However, competing priorities on nurse time could reduce the use of assessment tools to a tick box exercise, encourage blanket, rather than tailored, use of interventions and constrain nurse time with patients to personalise falls prevention plans. Previous studies have discussed similar impacts from systemising the documentation of care^18 35 36^ and have also shown how competing priorities can lead to staff feeling disempowered to implement falls prevention.^37^ Our findings indicated that interactions to help personalise care occurred outside the structured falls risk assessment, and that eliciting such information was supported by relatives and carers, who can provide a voice to express patients’ needs and concerns^38 39^ and non-clinical staff.

A clear theoretical divide can be made between falls risk screening and multifactorial falls risk assessment. Falls risk screening is not recommended for falls prevention in hospital, although hybrid approaches are seen in practice.^23^ In this study, risk screening, often to allocate enhanced patient supervision, was an established practice. While enhanced supervision may be necessary for some patients, this approach appears focused on mediating the effects of falls risks, for example, intervening to prevent a fall as it occurs, rather than modifying the underlying cause to reduce the risk of a fall. Like the use of non-slip socks, there is limited evidence to suggest supervision works to reduce falls incidents^40 41^; some studies discuss a conflict between the caring and restrictive aspects of this practice,^42 43^ and there are implications for patient privacy, recovery and discharge destination if it prevents or reduces patient mobilisation. Furthermore, these practices placed emphasis on nurses as responsible for preventing falls. Nurses’ key role in patient safety is well documented,^31 32^ but the multifactorial approach to falls prevention requires a multidisciplinary team response. Enhancing communication of patients’ individual falls risk factors across professional groups may help foster a greater sense of shared responsibility for falls prevention within the multidisciplinary team.^30 37^

### Strengths and weaknesses

This is the first empirical study to explore reasons for variation in the implementation of multifactorial falls risk assessment and personalised, multidomain prevention plans. A strength of this study is its use of realist evaluation, an iterative, theory-driven approach and multiple methods (observations, staff and patient carer interviews and EHR review). Data collection built on a robust foundation (hypotheses developed as a part of a realist review)^23^ and enabled the development of contextually based explanations that can support more reliable delivery of multifactorial falls prevention practices in hospitals. It has been noted that while guidelines tell us what to do, they say little about how these things should be done in practice.^44^ Therefore, the findings of this study have been translated into actionable guidance to support healthcare organisations to implement a multidisciplinary, personalised approach to fall prevention that focuses on the modification of individual falls risks.^45^

The four mechanisms tested reflect the complex and multifaceted nature of falls prevention. However, it would be beneficial to explore in greater detail multidisciplinary documentation systems and the delivery and impact of specific interventions intended to modify individual falls risks and the role and influence of organisational managers and teams on ward-based practices.

## Conclusion

To support reliable implementation of a multifactorial falls risk assessment and multidomain, personalised prevention plans, organisations should consider how established practices, including electronic systems and multidisciplinary communication, can be transformed to help ease the nursing burden and optimise the involvement of multidisciplinary teams and patients and carers in modification of individual falls risk factors. Consideration should be given to the resources and interactional skills needed to support patients with complex needs to participate in safety efforts related to falls prevention.

## Data Availability

Data are available on reasonable request.
