# Supplementary material for: Implementing recommended falls prevention practices for older patients in hospitals in England: a realist evaluation
Source: BMJ Open. 2025 Dec 14;15(12):e099698. doi: 10.1136/bmjopen-2025-099698 (PMC12706240; doi:10.1136/bmjopen-2025-099698)
Supplement: Supplementary file 1 [file bmjopen-2025-099698.reviewer_comments.pdf]

PEER REVIEW HISTORY

BMJ Open publishes all reviews undertaken for accepted manuscripts. Reviewers are asked to complete a checklist review form and are provided with free text boxes to elaborate on their assessment. These free text comments are reproduced below.

ARTICLE DETAILS

Title (Provisional)

Implementing recommended falls prevention practices for older patients in hospitals in England: a realist evaluation

Authors

Alvarado, Natasha; McVey, Lynn; Healey, Frances; Dowding, Dawn; Zaman, Hadar; Cheong, V-Lin; Gardner, Peter; Lynch, Alison; Hardiker, Nick; Randell, Rebecca

---

VERSION 1 - REVIEW

Reviewer 1

Name Naganathan, Vasi

Affiliation University of Sydney, Concord Clinical School, Faculty of Medicine and Health

Date 06-Apr-2025

COI None

This is an interesting study on a very important problem faced by hospitals around the world. The prevention of falls in hospitalised older people has been a "tough nut to crack". There have been some large well conducted trials that have directed interventions mainly at individual patients which have been negative. This has led to view that is important to look at this problem from a more "system" perspective. The importance of knowing more about the perspective of nurses and to understand more about the practicalities of delivering falls prevention intervention cannot be overstated.

This study adopts a realistic evaluation method. To the researchers' credit they have obtained 250 hours of observation, 40 staff interviews, 30 patient/carer interviews and review of 60 electronic health records. The conclusions reached is supported by the data obtained. It has relevance to not just falls prevention, but the management and prevention of other hospital acquired complications seen in older people such as delirium.

Please see attached PDF (place cursor on symbols) to see the comments. The following comment specific comments are intended to provide the editorial team and authors with suggestions with two main objectives:

- easier for a non-content expert to understand the manuscript

- more alignment with the latest literature

Title: accurate

Abstract: accurately reflects the study

Introduction:

this first paragraph does have "mixed sentences" suggest focus only on falls in hospital

give readers an idea of how big a problem this in the acute hospital setting in UK and costs...per 1000 bed days does not really do this. Many of the readers of BMC open may not really know why this is such an important issue

something like this: In UK hospitals, falls are a frequent safety concern, with over 240,000 reported annually, and 30-50% resulting in physical injury, including fractures in 1-3% of cases (with references)

Falls are the most frequently reported safety incident in NHS hospitals

Falls are estimated to cost the NHS ?

The evidence that multidomain interventions can prevent falls in acute hospital wards is not as strong as implied even if NICE recommends this. This needs to be acknowledged here and/or in discussion

The 2018 Cochrane review concluded: In hospitals: Multifactorial interventions may reduce rate of falls, although subgroup analysis suggests this may apply mostly to a subacute setting; we are uncertain of the effect of these interventions on risk of falling.

From 2022 World Falls Guidelines: A Cochrane review of 24 RCTs found that a multifactorial falls risk assessment, followed by implementation of multidomain interventions, may reduce the rate of falls in hospitals, but the very low-quality evidence precluded a definite conclusion. Reduction in falls rates was judged to be more likely in rehabilitation or geriatric ward settings

but did make this recommendation: Personalised single or multidomain falls prevention strategies based on identified risk factors, behaviours or situations should be implemented for all hospitalised older adults (≥65 years of age), or younger individuals identified by health professionals as at risk of falls. GRADE: 1C (Acute care), GRADE: 1B (Sub-acute care).

suggest: remove the 20-30% and keep the phrase "may reduce"

In the introduction or discussion worth saying this with references: intervention studies have shown that falls risk score removal does not impact inpatient falls. https://doi.org/10.1111/jgs.17125 and https://doi.org/10.1111/jocn.15471

Last paragraph: Make the “punchline” about the rationale for the study a standalone sentence: "The reasons for the variation have not been explored".

Methods:

Good description

Study setting: see minor edits. States that from three different organisations (because table 1 shows rows from 3 organisations)

Page 5, line 53: change subheading “study sample” to ”study sample and data collection”

Data collection would be easier to follow if this was numbered and the mechanism is mentioned again. Something like this

1. Leadership: what was done

2. Facilitation: what was done

3. Shared Responsibility: what was done?

4. Patient Participation: what was done

if there is overlap ie: the particular "data" obtained covers more than one mechanism perhaps a table would be a good way to summarise this. This is an important strength of this study, and the concern is readers will not fully appreciate this from just reading this paragraph as it stands

Analysis paragraph: “Data were compared against the mechanism to

understand if findings supported or refuted the explanation” -state what mechanism

Results:

clinical record review (page 7, line 60): change to EER so matches with wording in table 1

table 2 indicate that from 20 records from each organisation so that readers know what the denominator is without having to go back to table 1

Page 9, line 3: if prevention plans are individualised then add that fact to emphasise this point’’

The long sections” Established Falls Prevention practices” and “Competing Priorities on Nursing time” are hard to follow – can subheading and subthemes be used to make this easier? A table may help as well

Page 10, lines 37-24: However a challenge for nursing team……impact on them of delivering supervision:..”

This is a very an important point but could be "lost" when it comes under a paragraph about one risk factor for falls (medications). it is not as if the medication was stopped the risk of falls would have substantially decreased for many. This issue is important regardless of when on falls risk medications or not. See previous comment about subheading and subthemes and tables to help orientate the reader

Page 12, lines 20-22: “..rather than modifying the underlying cause…..these interventions” - should this sort of interpretation be in the discussion section?

Re non-slip socks: page 11, lines 50-52: in some organisations around the world there is a policy to not have non-slip socks: there is lack of evidence that they prevent falls and a thought it increases risk of skin damage

From Age and Ageing 2024: review of falls guidelines

https://doi.org/10.1093/ageing/afae149

Recent guidelines did not recommend the use of bed and chair alarms, non-slip socks and physical restraints [7, 41, 45]. Some of these interventions were mentioned in older clinical guidelines published before 2011 [51–54].

Page 12, lines 49-53: ‘The patient experience……” - good point but does not quite fit under Nursing priority’s theme

Discussion:

Strengths: mention something about the strong methodology (a number of different approaches) and the impressive numbers

Not sure that stating “restricted to nursing documentation” is a weakness because there were interviews with staff and patients/carers

Re the one the conclusions: “support patients with complex needs to participate in safety efforts related to falls prevention” – if this about patient/carer education then this point deserves it’s own paragraph in discussion and reference should be made to the evidence from the recent systematic reviews and the interventions studies that showed efficacy of falls education. The 2022 world guidelines for falls prevention (https://doi.org/10.1093/ageing/afac205 concluded: Strong recommendation. A tailored education on falls prevention should be delivered to all hospitalised older adults (≥65 years of age) and other high-risk groups. Grade 1A

check that that the evidence re interventions mentioned throughout is consistent with these two recent publications:

1. the Task Force on Global Guidelines for Falls in Older Adults , World guidelines for falls prevention and management for older adults: a global initiative, Age and Ageing, Volume 51, Issue 9, September 2022, afac205, https://doi.org/10.1093/ageing/afac205

2. Hospital falls clinical practice guidelines: a global analysis and systematic review, Age and Ageing, Volume 53, Issue 7, July 2024, afae149, https://doi.org/10.1093/ageing/afae149

The discussion could be strengthened with more references to current evidence and other studies that have explored the practicalities of implementing falls prevention interventions in the hospital setting. These references may provide support for the points be made in the discussion (that other studies have found findings that align with this study and people have made similar recommendations)

1. Preventing hospital falls: feasibility of care workforce redesign to optimise patient falls education. Age Ageing. 2024 Jan 2;53(1):afad250. doi: 10.1093/ageing/afad250. PMID: 38275097; PMCID: PMC10811524

2. Considerations across multiple stakeholder groups when implementing fall prevention programs in the acute hospital setting: a qualitative study. Age Ageing. 2024 Oct 1;53(10):afae208. doi: 10.1093/ageing/afae208. PMID: 39354814; PMCID: PMC11445322.

****** The reviewer provided a marked copy with additional comments. Please contact the publisher for full details. ******

**Reviewer**	**2**
**Name**	**Cavalcante, Tahissa**

**Affiliation**	**University of International Integration of Afro-Brazilian Lusophony**
**Date**	**26-Aug-2025**
**COI**	**None**

To the authors

The idea behind the study seems interesting, but it lacks a clear understanding of the issues and justification for conducting the study.

The study was conducted with a very small sample, which compromises the quality and generalizability of the results.

VERSION 1 - AUTHOR RESPONSE

Reviewer: 1
Prof. Vasi Naganathan, University of Sydney, Concord Repatriation General Hospital

Comments to the Author:
Introduction:
this first paragraph does have "mixed sentences" suggest focus only on falls in hospital give readers an idea of how big a problem this in the acute hospital setting in UK and costs...per 1000 bed days does not really do this. Many of the readers of BMC open may not really know why this is such an important issue something like this: In UK hospitals, falls are a frequent safety concern, with over 240,000 reported annually, and 30-50% resulting in physical injury, including fractures in 1-3% of cases (with references)
Falls are the most frequently reported safety incident in NHS hospitals. Falls are estimated to cost the NHS ?
In the introduction, we have added details about (and referenced) the number of falls incidents and injuries reported in hospitals in the UK, and the estimated cost to the NHS from inpatient falls, as suggested

The evidence that multidomain interventions can prevent falls in acute hospital wards is not as strong as implied even if NICE recommends this. This needs to be acknowledged here and/or in discussion. The 2018 Cochrane review concluded: In hospitals: Multifactorial interventions may reduce rate of falls, although subgroup analysis suggests this may apply mostly to a subacute setting; we are uncertain of the effect of these interventions on risk of falling. From 2022 World Falls Guidelines: A Cochrane review of 24 RCTs found that a multifactorial falls risk assessment, followed by implementation of multidomain interventions, may reduce the rate of falls in hospitals, but the very low-quality evidence precluded a definite conclusion. Reduction in falls

rates was judged to be more likely in rehabilitation or geriatric ward settings but did make this recommendation: Personalised single or multidomain falls prevention strategies based on identified risk factors, behaviours or situations should be implemented for all hospitalised older adults (≥65 years of age), or younger individuals identified by health professionals as at risk of falls. GRADE: 1C (Acute care), GRADE: 1B (Sub-acute care).suggest: remove the 20-30% and keep the phrase "may reduce"

We have prefaced the paragraph with '*the strength and quality of evidence is limited'* and removed 20 -30% from the sentence about fall reduction.

In the introduction or discussion worth saying this with references: intervention studies have shown that falls risk score removal does not impact inpatient falls. https://doi.org/10.1111/jgs.17125 and https://doi.org/10.1111/jocn.15471

We have added the sentence, '*studies have shown that discontinuing risk screening does not increase risk of falls'* in third paragraph of the introduction.

Last paragraph: Make the "punchline" about the rationale for the study a standalone sentence: "The reasons for the variation have not been explored".

This is now a standalone sentence.

Methods:
Study setting: see minor edits. States that from three different organisations (because table 1 shows rows from 3 organisations)
We have removed the last two sentence about number of hospitals as this is confusing to the reader. The main point is that three older person and three orthopaedic wards were studied in three organisations.

Page 5, line 53: change subheading "study sample" to "study sample and data collection"
We have changed the subheading as advised above and then deleted the separate heading for 'Data collection' below to reduce repetition.

Data collection would be easier to follow if this was numbered and the mechanism is mentioned again. Something like this
1. Leadership: what was done
2. Facilitation: what was done
3. Shared Responsibility: what was done?
4. Patient Participation: what was done
if there is overlap ie: the particular "data" obtained covers more than one mechanism perhaps a table would be a good way to summarise this. This is an important strength of this study, and the concern is readers will not fully appreciate this from just reading this paragraph as it stands

There is overlap as the reviewer suggests – observations and interviews were used to test all mechanisms. We have reworded the paragraph to clarify this point be explicitly referring to Leadership, Facilitation, Shared Responsibility, and Patient Participation and providing examples of how prompts in the observation schedule related to different mechanisms.

Analysis paragraph: "Data were compared against the mechanism to understand if findings supported or refuted the explanation" -state what mechanism

We have listed - Leadership, Facilitation, Shared Responsibility, and Patient Participation to be explicit about the content of each framework.

Results:
clinical record review (page 7, line 60): change to EER so matches with wording in table 1

Changed to EHR (Electronic Health Record).

table 2 indicate that from 20 records from each organisation so that readers know what the denominator is without having to go back to table 1

'Per-site, n=20' has been added to the assessment and prevention plan column header.

Page 9, line 3: if prevention plans are individualised then add that fact to emphasise this point''

This section discusses risk items included in the assessment documentation. We have amended the first sentence to read – 'Table 3 demonstrates that falls risk assessment documentation included multiple risks, but the extent to which they explicitly encompassed the NICE domains as *prompts in the assessment and care plan differed*'.

The long sections" Established Falls Prevention practices" and "Competing Priorities on Nursing time" are hard to follow – can subheading and subthemes be used to make this easier? A table may help as well

Thank you for this helpful comment. We have added explanation under the main themes (Established falls prevention practices and Competing priorities) to clarify that they represent contextual influences on the mechanisms under investigation. We have also added sub- headings to separate the findings by mechanism.

Page 10, lines 37-24: However a challenge for nursing team……impact on them of delivering supervision:.."
This is a very an important point but could be "lost" when it comes under a paragraph about one risk factor for falls (medications). it is not as if the medication was stopped the risk of falls would have substantially decreased for many. This issue is important

regardless of when on falls risk medications or not. See previous comment about subheading and subthemes and tables to help orientate the reader

We have reconfigured these findings under two sub-headings (1) Ward leadership for falls prevention, and (2) Enhanced patient supervision

Page 12, lines 20-22: "..rather than modifying the underlying cause.....these interventions" - should this sort of interpretation be in the discussion section?

We have now moved and integrated this reflection into the third paragraph of the discussion.

Re non-slip socks: page 11, lines 50-52: in some organisations around the world there is a policy to not have non-slip socks: there is lack of evidence that they prevent falls and a thought it increases risk of skin damage
From Age and Ageing 2024: review of falls guidelines
https://doi.org/10.1093/ageing/afae149 Recent guidelines did not recommend the use of bed and chair alarms, non-slip socks and physical restraints [7, 41, 45]. Some of these interventions were mentioned in older clinical guidelines published before 2011 [51–54].

We agree that there is a lack of evidence re. non-slip socks. We have amended the first paragraph of the discussion and added the following sentence to acknowledge the lack of evidence to support their use – 'In the following discussion, it should be noted that in addition to exploring why aspects of care recommended in clinical guidelines were not implemented, we also observed many practices embedded in care which guidelines find little or no evidence to support such as non-slip socks.

Page 12, lines 49-53: 'The patient experience......" - good point but does not quite fit under Nursing priority's theme

We have added a sentence under the main heading to explain that competing priorities and workload pressure influenced (1) nurse use of the falls risk assessment documentation in the EHR and (2) how and the extent to which patients participated in falls prevention.

We have added 'Patient participation in falls prevention planning' as a subheading and reconfigured this section to clarify that competing priorities and workload pressure constrained patient participation in (1) falls risk assessment and prevention planning and (2) interventions in which they were commonly encouraged to participate such as use of the call bell and rehabilitation programmes.

Discussion:
Strengths: mention something about the strong methodology (a number of different approaches) and the impressive numbers
Not sure that stating “restricted to nursing documentation” is a weakness because there were interviews with staff and patients/carers

We have amended the strengths and weaknesses section to read: ‘A strength of this study is its use of realist evaluation, an iterative, theory driven approach, and multiple methods (observations, staff and patient carer interviews, clinical record review). Data collection built on a robust foundation (hypotheses developed as part of a realist review) and enabled the development of contextually based explanations that can support more reliable delivery of multifactorial falls prevention practices in hospitals. The four mechanisms tested reflect the complex and multifaceted nature of falls prevention.

Re the one the conclusions: “support patients with complex needs to participate in safety efforts related to falls prevention” – if this about patient/carer education then this point deserves it’s own paragraph in discussion and reference should be made to the evidence from the recent systematic reviews and the interventions studies that showed efficacy of falls education. The 2022 world guidelines for falls prevention (https://doi.org/10.1093/ageing/afac205 concluded: Strong recommendation. A tailored education on falls prevention should be delivered to all hospitalised older adults (≥65 years of age) and other high-risk groups. Grade 1A

This conclusion relates to how competing priorities on staff time constrain patient participation, which is discussed in paragraph two of the discussion.

check that that the evidence re interventions mentioned throughout is consistent with these two recent publications:
1. the Task Force on Global Guidelines for Falls in Older Adults , World guidelines for falls prevention and management for older adults: a global initiative, Age and Ageing, Volume 51, Issue 9, September 2022, afac205, https://doi.org/10.1093/ageing/afac205
2. Hospital falls clinical practice guidelines: a global analysis and systematic review, Age and Ageing, Volume 53, Issue 7, July 2024, afae149, https://doi.org/10.1093/ageing/afae149

Thank you for highlighting these papers. The interventions of interest in this study were multifactorial falls risk assessments and multidomain prevention plans, use of which are consistent with these publications.

Specific interventions are discussed in the findings e.g., enhanced supervision and non-slip socks. These interventions were observed by researchers and discussed

by interviewees but have limited evidence to support effectiveness. In the discussion we highlight that non-slip socks and supervision have limited evidence supporting their use in falls prevention in hospital.

The discussion could be strengthened with more references to current evidence and other studies that have explored the practicalities of implementing falls prevention interventions in the hospital setting. These references may provide support for the points be made in the discussion (that other studies have found findings that align with this study and people have made similar recommendations)

1. Preventing hospital falls: feasibility of care workforce redesign to optimise patient falls education. Age Ageing. 2024 Jan 2;53(1):afad250. doi: 10.1093/ageing/afad250. PMID: 38275097; PMCID: PMC10811524
2. Considerations across multiple stakeholder groups when implementing fall prevention programs in the acute hospital setting: a qualitative study. Age Ageing. 2024 Oct 1;53(10):afae208. doi: 10.1093/ageing/afae208. PMID: 39354814; PMCID: PMC11445322.

Thank you for highlighting these studies. We have now referenced the second paper that supports and enhances our findings related to shared responsibility, competing priorities and workload pressure.

Reviewer: 2
Dr. Tahissa Cavalcante, University of International Integration of Afro-Brazilian Lusophony

Comments to the Author:
To the authors
The idea behind the study seems interesting, but it lacks a clear understanding of the issues and justification for conducting the study.

The issue we have investigated is variation in the implementation of falls prevention practices recommended by the National Institute for Health and Care Excellence (NICE). NICE is a UK organization that provides national, evidence-based guidance and advice to improve health and social care and reduce disparities in treatment availability. The key elements of these practices (multifactorial risk assessment linked to multidomain, personalised interventions) are explained in the introduction.

The study rationale is explained in the introduction – that despite recommendations about multifactorial falls prevention practices, there is variation in how they are delivered, reasons for which have not been explored, and falls

remain a persistent safety concern. In response to both reviewers' helpful comments, we have added more sources that emphasise the scale of harm from falls in hospitals and the gap between evidence and practice in specific aspects of falls prevention (see responses to Reviewer 1).

The study was conducted with a very small sample, which compromises the quality and generalizability of the results.
To the best of our knowledge, this is the first and the largest empirical study of implementation of multifactorial falls prevention practices in hospitals. Our analyses include data from 251 hours of observation of care, 40 staff interviews, 31 patient and carer interviews, and a review of 60 clinical records. The case study design and theory driven approach enabled us to investigate how contextual factors influenced mechanisms theorised to underpin implementation of the practices of interest. The mechanisms were prioritised for investigation by stakeholders, including patient and public representatives. This approach supports analytic rather than statistical generalisability.

VERSION 2 - REVIEW

Reviewer **1**

Name **Naganathan, Vasi**

Affiliation **University of Sydney, Concord Clinical School, Faculty of Medicine and Health**

Date **09-Nov-2025**

COI

---

the manuscript is now easier to follow.

no other suggestion

I have no concerns about the size of the study. the numbers are impressive for this kind of study
